# BPTF in bone marrow provides a potential progression biomarker regulated by TFAP4 through the PI3K/AKT pathway in neuroblastoma

**DOI:** 10.1186/s12575-023-00200-7

**Published:** 2023-05-11

**Authors:** Chiyi Jiang, Yeran Yang, Sidou He, Zhixia Yue, Tianyu Xing, Ping Chu, Wenfa Yang, Hui Chen, Xiaoxi Zhao, Yongbo Yu, Xuan Zhang, Yan Su, Yongli Guo, Xiaoli Ma

**Affiliations:** 1 Medical Oncology Department, Pediatric Oncology CenterNational Center for Children’s HealthKey Laboratory of Pediatric Hematology Oncology, Key Laboratory of Major Diseases in Children, Ministry of Education, Beijing Children’s Hospital, Capital Medical University, 56 Nanlishi Road, Beijing, Xicheng District China; 2 Beijing Key Laboratory for Pediatric Diseases of Otolaryngology, Head and Neck Surgery, MOE Key Laboratory of Major Diseases in Children, Beijing Pediatric Research Institute, Beijing Children’s Hospital, Capital Medical University, National Center for Children’s Health, 56 Nanlishi Road, Beijing, Xicheng District China; 3 Hematologic Disease LaboratoryKey Laboratory of Pediatric Hematology OncologyNational Key Discipline of Pediatrics (Capital Medical University)Key Laboratory of Major Diseases in Children, Ministry of Education, Beijing Pediatric Research Institute, Beijing Children’s Hospital, Capital Medical University, National Center for Children’s Health, Hematology Center, Beijing, China

**Keywords:** BPTF, Bone marrow, Neuroblastoma, TFAP4, PI3K/AKT

## Abstract

**Background:**

Neuroblastoma (NB) is the most common extracranial malignant solid tumor in children, which is highly prone to bone marrow (BM) metastasis. BM can monitor early signs of mild disease and metastasis. Existing biomarkers are insufficient for the diagnosis and treatment of NB. Bromodomain PHD finger transcription factor (BPTF) is an important subunit of the chromatin-remodeling complex that is closely associated with tumors. Here, we evaluated whether BPTF in BM plays an important role in predicting NB progression, and explore the molecular mechanism of BPTF in NB.

**Methods:**

The clinical relevance of the BPTF was predicted in the GEO (GSE62564) and TARGET database. The biological function of BPTF in NB was investigated by constructing cell lines and employing BPTF inhibitor AU1. Western blot was used to determine the changes of BPTF, TFAP4, PI3K/AKT signaling and Epithelial-mesenchymal transition (EMT) related markers. A total of 109 children with newly diagnosed NB in Beijing Children's Hospital from January 2018 to March 2021 were included in this study. RT-PCR was used to measure the BPTF and TFAP4 expression in BM. The cut-off level was set at the median value of BPTF expression levels.

**Results:**

Databases suggested that BPTF expression was higher in NB and was significantly associated with stage and grade. Proliferation and migration of NB cells were slowed down when BPTF was silenced. Mechanistically, TFAP4 could positively regulate BPTF and promotes EMT process through activating the PI3K/AKT signaling pathway. Moreover, detection of the newly diagnosed BM specimens showed that BPTF expression was significantly higher in high-risk group, stage IV group and BM metastasis group. Children with high BPTF at initial diagnosis were considered to have high risk for disease progression and recurrence. BPTF is an independent risk factor for predicting NB progression.

**Conclusions:**

A novel and convenient BPTF-targeted humoral detection that can prompt minimal residual and predict NB progression in the early stages of the disease were identified. BPTF inhibitor AU1 is expected to become a new targeted drug for NB therapy. It’s also reveal previously unknown mechanisms of BPTF in NB cell proliferation and metastasis through TFAP4 and PI3K/AKT pathways.

**Supplementary Information:**

The online version contains supplementary material available at 10.1186/s12575-023-00200-7.

## Background

Neuroblastoma (NB) is the most common extracranial solid tumor in infants and young children, with an incidence rate of 10.2 cases/per million under 15 years old [1]. As one of the most malignant of the neuroblastic tumor, it is characterized by insidious onset and prone to metastasis of bone marrow (BM), bones as well as distant organs [2]. About 50% of children with high-risk (HR) NB will also progress or relapse during treatment, although the application of immunization and targeted drugs has increase in recent years [3, 4]. The treatment of these children is difficult and the long-term prognosis is extremely poor [5]. Therefore, exploring the causes of NB progression and the relevant molecular mechanisms to improve prognosis is the focus of current study.

As a common site of NB metastasis, BM provides an accessible and minimally invasive liquid sample by which to monitor early signs of minor disease and metastasis [6]. At present, the main biomarkers of NB detected by BM are *MYCN* amplification, 11q23 and 1p36 loss, *PHOX2B* expression, etc. [7, 8]. Although these markers are strongly associated with poor prognosis NB, they does not predict all cases of poor survival in NB [9]. Given the advantages of readily available liquid biopsy, it is urgent to find new bio-targeted molecules in BM for children with NB.

Bromodomain PHD finger transcription factor (BPTF), the core subunit of the nucleosome remodeling factor NURF. It can recruit other subunits of the NURF complex to the promoter or enhancer region of downstream genes, and promote these genes’ transcription by regulating nucleosome sliding [10]. Several studies reported that BPTF was closely related to the occurrence and development of tumors, such as hepatocellular carcinoma [11] and lung adenocarcinoma [12]. However, the relationship between BPTF and NB has not been reported. Meanwhile, the functions and molecular mechanisms of BPTF in NB proliferation and metastasis remain to be elucidated.

Transcriptional factor activating protein 4 (TFAP4) belongs to the leucine zipper family and is widely involved in tumor proliferation, differentiation, metastasis, angiogenesis and other biological regulatory functions [13]. According to reports, TFAP4 was significantly increased in NB children with *MYCN* amplification [14]. And TFAP4 may be an important element in the positive feedback loop that maintains the activation of the PI3K/AKT pathway in prostate cancer [15]. However, the molecular mechanism between TFAP4 and BPTF in NB is unclear.

Our previous research found that the somatic mutation of *BPTF* gene was positively correlated with NB tumor burden by whole gene exome sequencing [16]. Inspired by that, this study aimed to reveal BPTF as a novel target in the BM to predict NB progression through clinical relevance analysis, functional studies, and the molecular mechanism exploration of TFAP4 involved in BPTF carcinogenesis.

## Materials and methods

### Patients and samples

There were 109 BM samples obtained from newly diagnosed children with NB. All participants were firstly diagnosed with NB in the Medical Oncology Department of Beijing Children’s Hospital, Capital Medical University (BCH) from January 2018 to March 2021. They all received regular treatments with chemotherapy (BCH-NB-2007 protocol), surgery, transplantation, radiotherapy and oral retinoic acid. All patients were staged according to the International Neuroblastoma Stage System (INSS) [17]. Risk stratification was conducted according to the Children’s Oncology Group (COG) [18]. Children in the HR group met the following conditions: age > 18 months, with distant metastasis or *MYCN* gene amplification. Tumor status in the BM was performed using BM cytomorphologic examination and *PHOX2B* test. Clinical information such as age, gender, primary tumor site, metastatic site, gene characteristics and tumor markers were retrospectively collected. The enrolled children were followed up until September 30, 2021. The other 16 BM samples from minimal residual disease (MRD)-negative children who had been completely remission (CR) for more than 3 years were regarded as control cohorts [19]. Informed consent were acquired from all participants and their parents. The study was approved by the Ethics Committee of the BCH (2019-k-42).

### Cell culture

Human NB cell line SK-N-BE (2) and SH-SY5Y were purchased from American type culture collection (ATCC, USA), and guaranteed to be used within 6 months after resuscitation. It is routinely cultured in DMEM medium containing 10% fetal bovine serum (Corning, USA) at 37℃ 5% CO2 incubator (Thermo Fisher Scientific, USA). BPTF inhibitor AU1 (GlpBio, USA) was administered at different concentrations of 20–40 μM [20].

### Lentiviral infection and transfection of siRNA

Lentiviral of shRNA with GFP-tag and plasmid of overexpressing the full-length TFAP4 were synthesized by Shanghai Genepharma Company (China). Their sequences were: shNC: 5’-TTCTCCGAACGTGTCACGT-3’; shBPTF-1: 5’-CAGGAGAGTTCTCAAGTAG-3’; shBPTF-2: 5’-GGTGGCAATCAAGGTTTGA-3’. Cells were harvested 96 h after infection for related experiments. siRNAs were synthesized by Guangzhou RiboBio Company (China). The sequences used were as follows: siNC: 5’-GGCUCUAGAAAAGCCUAUGC-3’; siTFAP4-1: 5’-TGGGATTGTCAGCCTTCAA-3’; siTFAP4-2: 5’-GGACAAGGACGAAGGCATA-3’. Transfection was performed following the instruction of Lipofectamine RNAiMax (Thermo Fisher Scientific, USA) when the cell confluence in the 6-well culture plate was 30–50%. After 72 h, cells were collected and the subsequent experiments were done.

### Establish BPTF stable knockout cell line by CRISPR/Cas9

To establish a BPTF knock-out (KO) stable cell line in SK-N-BE (2), the sequence of sgRNA-BPTF was 5’-TTTACGAGGTACTGCGGAAC-3’ belonged to the exon 2 [21]. BPTF CRISPR/Cas9 KO sgRNA lentiviral and Control (Scramble) CRISPR/Cas9 sgRNA lentiviral were purchased from Shanghai Genepharma Company (China). After 96 h of infection, cells were plated into 10 cm dishes at different concentrations from 1/50 to 1/2000. Single-cell-derived surviving colonies were manually picked and individually transferred into a 24-well plate after about 3 weeks. BPTF-KO cell lines were screened by PCR and sequencing, and validated by western blot.

### Real-time PCR (RT-PCR)

Total RNA from cell lines and NB BM specimens was extracted with TRIzol reagent (Invitrogen, US) according to the instructions. cDNA synthesis was performed using the RNA reverse transcription kit (Invitrogen, US). RT-PCR was accomplished using ABI 7100 RT-PCR Instrument (Applied Biosystems, Singapore) with SYBR-Green (Invitrogen, US). The sequence of primers were as follows: GAPDH (Forward: 5’-TGCACCACCAACTGCTTAG-3’, Reverse: 5’-GATGCAGGGATGATGTTC-3’); BPTF (Forward: 5’-TCCACACGAGACAAAGTGAAAC-3’, Reverse: 5’-AAAAATGCTCTTCTTGCTGCTC-3’); TFAP4 (Forward: 5′-GTGCCCACTCAGAAGGTGC-3′, Reverse: 5′-GGCTACAGAGCCCTCCTATCA-3′). The reaction condition of RT-PCR was as follows: 95 °C for 10 min, 95 °C for 15 s and 60 °C for 1 min, totaling 40 cycles.

### Western blot analysis

Protein samples were extracted using RIPA lysis buffer (Thermo Fisher Scientific, USA) containing a protease inhibitor cocktail and a phosphatase inhibitor cocktail (Roche, Germany). The protein concentration was mensurated by Pierce BCA Protein Assay Kit (Thermo Fisher Scientific, USA) according to the manufacturer's instructions. A total of 15–20 μg protein was separated in 4–12% precast glue (cat. HLG2001-412 T, Shanghai Haling Biotechnology, China) or 10% sodium dodecyl sulfate–polyacrylamide gel electrophoresis (SDS-PAGE), and transferred to 0.45 μm PVDF membrane (Millipore, Germany). The antibodies anti-BPTF (cat. ab72036, 1:2000), and anti-N-cadherin (cat. ab76057, 1:1000) were from Abcam (Cambridge, UK); anti-TFAP4 (cat. Sc-377042, 1:1000) and anti-E-cadherin (cat. sc-8426, 1:1000) were from Santa Cruz Biotechnology (Santa Cruz, USA); anti-AKT (cat. CST-4691t, 1:1000), anti-pAKT (cat. CST-13038S, 1:1000) and anti-GAPDH (cat. CST-5174S, 1:1000) were from Cell Signaling Technology (Danvers, USA); anti-HA (cat. H9658 1:20,000) was from Sigma (St. Louis, USA). The primary antibody was combined at 4℃ overnight and anti-rabbit/mouse HRP (Proteintech, USA) at 1:3000 dilutions for about 1 h at room temperature. The western blotting band was observed by enhanced chemiluminescence reagents (Thermo Fisher Scientific, USA).

### Immunohistochemistry (IHC)

The fixed tumor specimens were embedded in paraffin and sectioned. Unstained 4 µm FFPE sections were heated with 10 mM sodium citrate buffer (pH 6.0) for 15 min at 95 °C to retrieve tissue antigen after dewaxing. Then the sections were incubated with 3% hydrogen peroxide for 10 min at room temperature. The diluted primary antibody of anti-Ki67 (Abcam, cat. ab15580, 1:1000) were added to each section and incubated overnight at 4 °C. The secondary antibody kit (cat. PV‐9001) were purchased from Zhongshan Golden Bridge Biotechnology (Beijing, China). Staining was evaluated independently to determine the histological score according to the proportion of positive staining cells and intensity as described previously [22].

### Real-time cellular analysis

Cell proliferation and migration experiments were performed according to the instruction of xCELLigence Real-Time Cell Analysis Dual Purpose (RTCA DP) (ACEA Biosciences, USA). To monitor cell proliferation, 50 μl medium was added to each well of E-Plate 16 to detect baseline. The Cell Index in all wells was below 0.063. After detecting the cell concentration, 100 μl cell suspension with 4 × 10^3^ cells were added to each well. To perform migration experiment, 165 μl DMEM medium with 10% FBS was added to the CIM lower chamber, meanwhile 30 μl FBS-free DMEM medium was added to the upper chamber. The baseline was detected after balancing the installed CIM-Plate in the 37℃ incubator for 1 h. And then 100 μl FBS-free DMEM medium with 4 × 10^4^ cells were added to each upper chamber. Four replicate wells were set in each group. After standing in the 37℃ incubator for 30 min, real-time dynamic monitoring was started. The cell index was measured every 15 min. The test lasted for at least 96 h for proliferation experiment and at least 48 h for migration test.

### Colony formation assay

Cells were seeded into 6-well plates (~ 800 cells/well) in triplicate and were further incubated in a complete medium for 14 days. Colonies were fixed with 4% paraformaldehyde and were stained with 0.1% crystal violet. After being washed with PBS, the colonies were photographed and calculated.

### Transwell cell invasion assay

Matrigel-coated (Coning, USA) and transwell inserts (8 μm pore size, BD Falcon, USA) were used to evaluate cell migration and invasion. A total of 350 μl FBS-free medium with 1 × 10^5^ cells were added to the upper chambers. The lower chamber contained an 800 μl medium with 10% FBS. After being incubated for 24 h, the cells on the upper surface of the films were removed by cotton swabs gently. The migrated cells of the lower surface were sequentially fixed with 4% paraformaldehyde, stained with crystal violet for 15 min respectively, and washed in PBS. Cells undergoing invasion were imaged and counted in 5 fields of view.

### Wound healing assay

Cells were seeded in 6-well plates and grown to nearly 80–100% confluence. A straight line was drawn across the plate with a pipette tip. The monolayer was washed with an FBS-free medium to remove detached cells, and 2% FBS medium was added. The images of the cells were photographed at 0 h, 12 h, and 24 h post-wounding.

### Cell apoptosis and cell cycle assay

Cell apoptosis was detected using an Annexin V-APC/7-AAD staining kit and the cell cycle was tested using PI detection kit (KeyGEN BioTECH, China) according to the manufacturer's protocol by flow cytometry.

### In vivo studies

All animal experiments were conducted according to the guidelines of the Animal Experiments and Experimental Animal Welfare Committee of Capital Medical University. Ten female BALB/c nude mice (aged 4 weeks, 18–22 g) were randomly divided into 2 groups (DMSO, AU1). SK-N-BE (2) cells (5 × 10^6, 100 μL in serum-free DMEM 50% matrigel) were subcutaneously implanted. AU1 was administrated at a dose of 15 mM every 3 days intratumorally beginning at the tumor volume reached 150–400 mm^3^. The growth of tumors was monitored every 3 days. The tumor volume was calculated using the formula volume = (length × width^2)/2. After 12 days, the mice were sacrificed by cervical dislocation. Tumor were fetched, weighed, and fixed in 10% formalin for histological analysis.

### Statistics

Each experiment was repeated at least three times. Experimental data were expressed as mean ± standard error (SEM) or standard deviation (SD). The differences between groups were analyzed using Student’s *t*-test.

Children were split into high and low BPTF expression groups based on the median values of BPTF expression. Mann–Whitney rank and chi-square test were applied to compare clinical characteristics and tumor biomarkers between the two groups. The Kaplan–Meier method was used to estimate Event-free survival (EFS) and Overall survival (OS), which were regarded as relapse-progression outcome indicator. Group difference was calculated with the log-rank test. The correlation between genetic variables were determined by Spearman’s test. Univariate and multivariate COX regression were conducted to analyse the risk factors for EFS. Hazard ratios (HR) and corresponding 95% confidence interval (CI) were reported. The statistical analysis was performed using SPSS version 22 (SPSS Inc, Chicago, IL, USA), GraphPad Prism version 8 (GraphPad Software, San Diego, CA, USA), Origin version 2022 (OriginLab Corporation, Northampton, MA, USA) and Figdraw. A *P*-value < 0.05 was considered statistically significant.

## Results

### Higher BPTF is associated with poor survival and high malignancy in NB through databases analysis

**Fig. 1:**
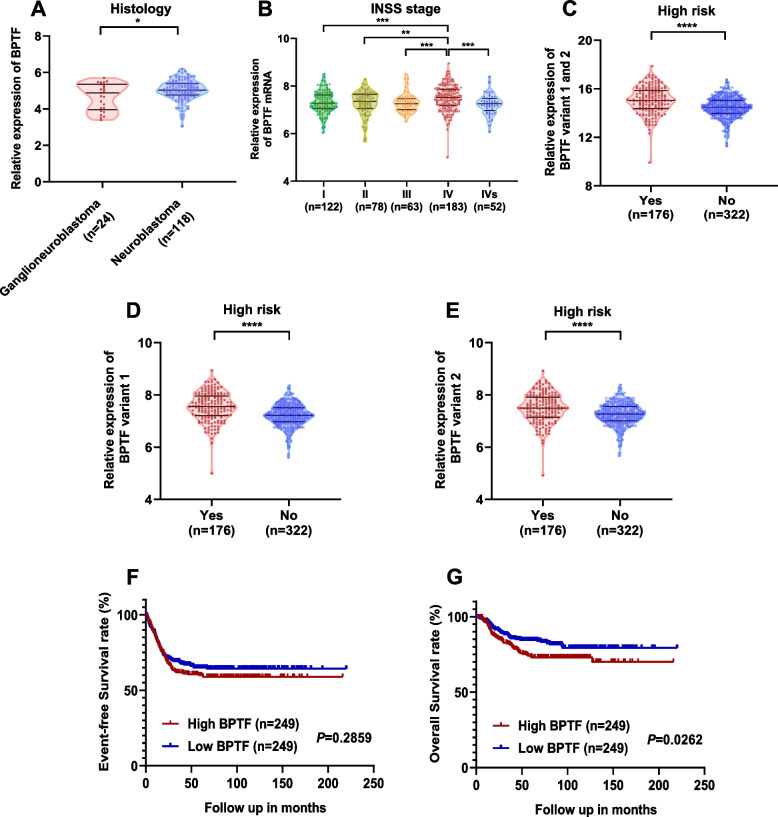
Higher BPTF is associated with poor survival and high malignancy in NB through databases analysis. **A** The TARGET database suggests that BPTF is highly expressed in NB tissues than in ganglioneuroblastoma [median, 5.038 vs. 4.882, *n* = 142]. **B** The GEO database suggests that BPTF is highly expressed in stage IV than in other stage [median, 7.510 vs. 7.284, *n* = 498]. **C**-**E** The BPTF expression of the different transcripts was higher in high-risk group than in low-risk group from the GEO database [median, 15.047 vs. 14.489, 7.553 vs. 7.224, 7.499 vs. 7.271, *n* = 498]. **F**-**G** Poor prognosis of children with NB in high BPTF group (*n* = 498). *P* = 0.2859 [5-year EFS, 60.0% vs. 64.9%, F], *P* = 0.0262 [5-year OS, 73.6% vs. 84.5%, G], by log-rank test. * *P* < 0.05, ** *P* < 0.01, **** *P* < 0.0001, as assessed by Mann Whitney test

Given that previous studies demonstrated a positive correlation of BPTF mutation rate with NB primary focal tumor volume, bioinformatics analysis was performed to investigate the role of BPTF in NB progression. Analysis of the 142 cohorts obtained from the TARGET database (https://target-data.nci.nih.gov/Public/NBL/clinical/) revealed that the expression level of BPTF mRNA were higher in NB than in ganglioneuroblastoma [median, 5.038 vs. 4.882, *P* = 0.046, Fig. 1A], which was one of the neuroblastic tumor with less malignancy than NB. Data from 498 NB patients in the GEO dataset (GSE62564) suggested that the expression level of BPTF in children at stage IV was significantly higher than that in children at non-stage IV [median, 7.510 vs. 7.284, *P* < 0.0001, Fig. 1B]. Moreover, the expression of BPTF in children with NB in the HR group was significantly higher than that in the low/intermediate-risk group [median, 7.553 vs. 7.224, *P* < 0.0001, Fig. 1C-E]. In addition, the prognosis of children with high BPTF was worse [5-year EFS, 60.0% vs. 64.9%, *P* = 0.2859, Fig. 1F; 5-year OS, 73.6% vs. 84.5%, *P* = 0.0262, Fig. 1G].

### BPTF exerts a promotional role in NB cell proliferation

**Fig. 2:**
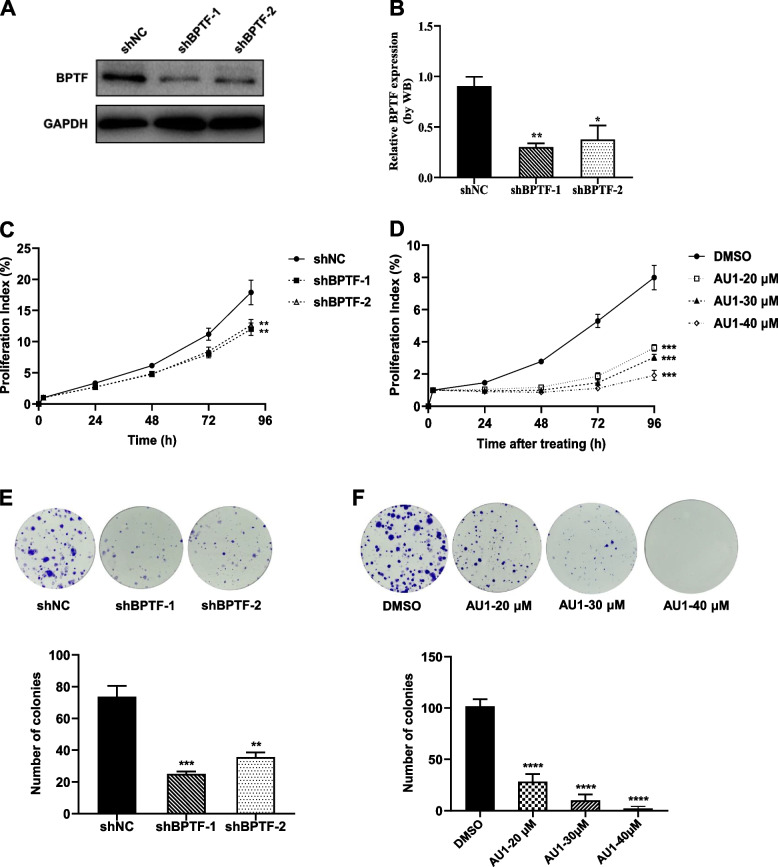
BPTF exerts a promotional role in neuroblastoma cell growth. **A** Representative expression of BPTF after 96 h infection with BPTF knockdown lentiviral, as detected by western blot. **B** BPTF western blot analysis in SK-N-BE(2) cell [mean, 0.904 vs. 0.302 vs. 0.377]. **C**-**D** SK-N-BE(2) cell growth following BPTF depletion (**C**) and using AU1 (**D**) shown by RTCA. Error bars represent SD. **E**–**F** SK-N-BE(2) cell growth following BPTF depletion [mean, 73.667 vs. 25.000 vs. 35.667, E] and using AU1 [mean, 101.667 vs. 28.333 vs. 10.000 vs. 2.000, F] shown by colony formation assay. Error bars represent SEM. The data are representative of at least three independent experiments. ** *P* < 0.01, *** *P* < 0.001, **** *P* < 0.0001, as assessed by Student’s *t*-tests

To investigate whether NB depends on BPTF for cell proliferation, SK-N-BE (2) cells were transfected with lentiviral shBPTF and shNC (Fig. S1A). All subsequent experiments for depletion of BPTF were performed at this time point. RT-PCR and western blot were used to detect the knock-down efficiency on mRNA and protein levels, indicating that both shBPTF were effective and the shBPTF-1 efficiency was higher (Fig. 2A-B, Fig. S1B). In addition, BPTF stable knockout cell lines also established by CRISPR/Cas9 in SK-N-BE (2) and SH-SY5Y cells (Fig. S1C-H). The results showed that depletion of BPTF significantly reduced NB cell proliferation (Fig. 2C) and colony formation (Fig. 2E, Fig. S2A). Furthermore, SK-N-BE (2) cell colony formation was significantly reduced in present at AU1, which is the inhibitor of BPTF (Fig. 2D, F). The above results showed that BPTF could promote the proliferation of NB cells and it exerted a promotional role in NB growth.

### BPTF leads to increased migration and invasion in NB cells

**Fig. 3:**
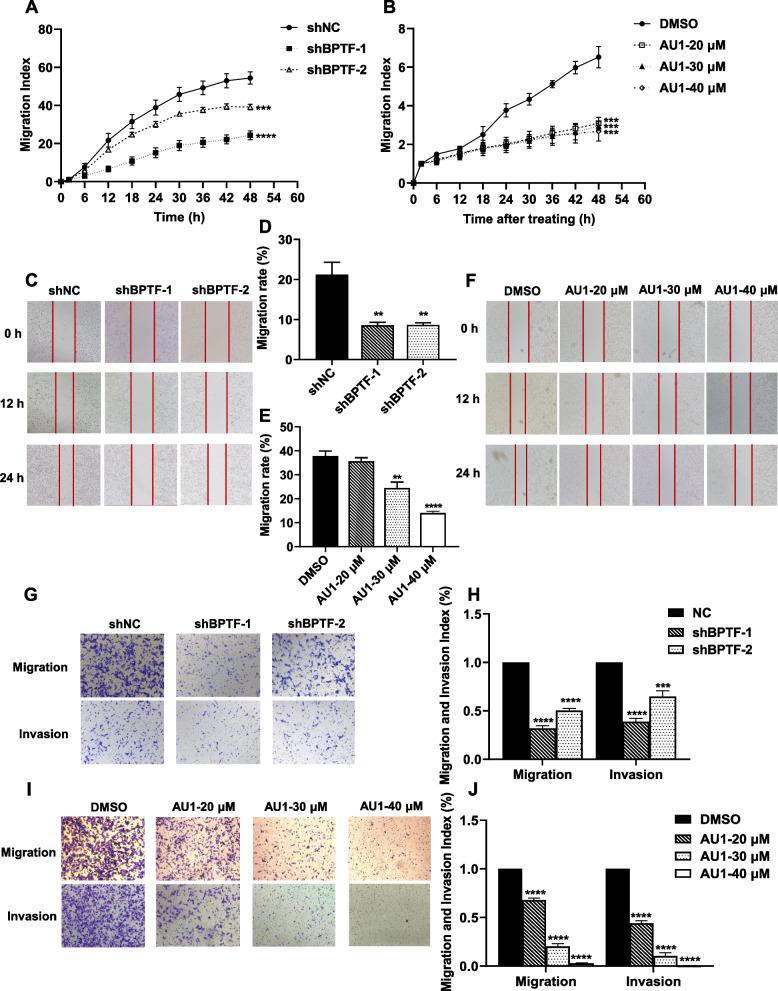
BPTF leads to an increase in neuroblastoma cell migration and invasion. **A**-**B** SK-N-BE (2) cell migration following BPTF depletion (**A**) and using AU1 (**B**) shown by RTCA. Error bars represent SD. **C**-**F** Wound healing assay(0 h, 12 h, 24 h) to observe the migration of SK-N-BE (2) cell line and wound healing rates 24 h after scratch following BPTF knockdown [mean, 21.2% vs. 8.6%vs. 8.6%, C-D] and using AU1 [mean, 37.9% vs. 35.6% vs. 24.4% vs. 14.0%, E–F]. **G**-**J** Compare the invasion and migration of SK-N-BE (2) cell following BPTF downregulation [mean, 1.000 vs. 0.319 vs. 0.503, G; 1.000 vs. 0.387 vs. 0.648, H] and using AU1 by transwell [mean, 1.000 vs. 0.679 vs. 0.203 vs. 0.028, I; 1.000 vs. 0.441 vs. 0.105 vs. 0.005, J]. Error bars represent SEM. The data are representative of at least three independent experiments. ** *P* < 0.01, *** *P* < 0.001, **** *P* < 0.0001, as assessed by Student’s *t*-tests

Database analysis hints that BPTF is involved in migration and invasion in NB cells. To explore the role of BPTF on cellular migration in NB cells, RTCA technology, wound healing assay and transwell assay without Matrigel-coated were performed. It revealed that depletion of BPTF significantly reduced migration rate in SK-N-BE (2) and SH-SY5Y cells (Fig. 3A, C-D, G-H, Fig. S2B). Meanwhile, applying transwell chambers covered with Matrigel, it showed that knockdown of BPTF expression decreased the invasion number and rate of SK-N-BE (2) cells (Fig. 3G-H). Similarly, migration and invasion rates were significantly inhibited with the BPTF inhibitor AU1 (Fig. 3B, E–F, I-J).

### BPTF deficiency induces apoptosis of NB cells and causes mitotic arrest

**Fig. 4:**
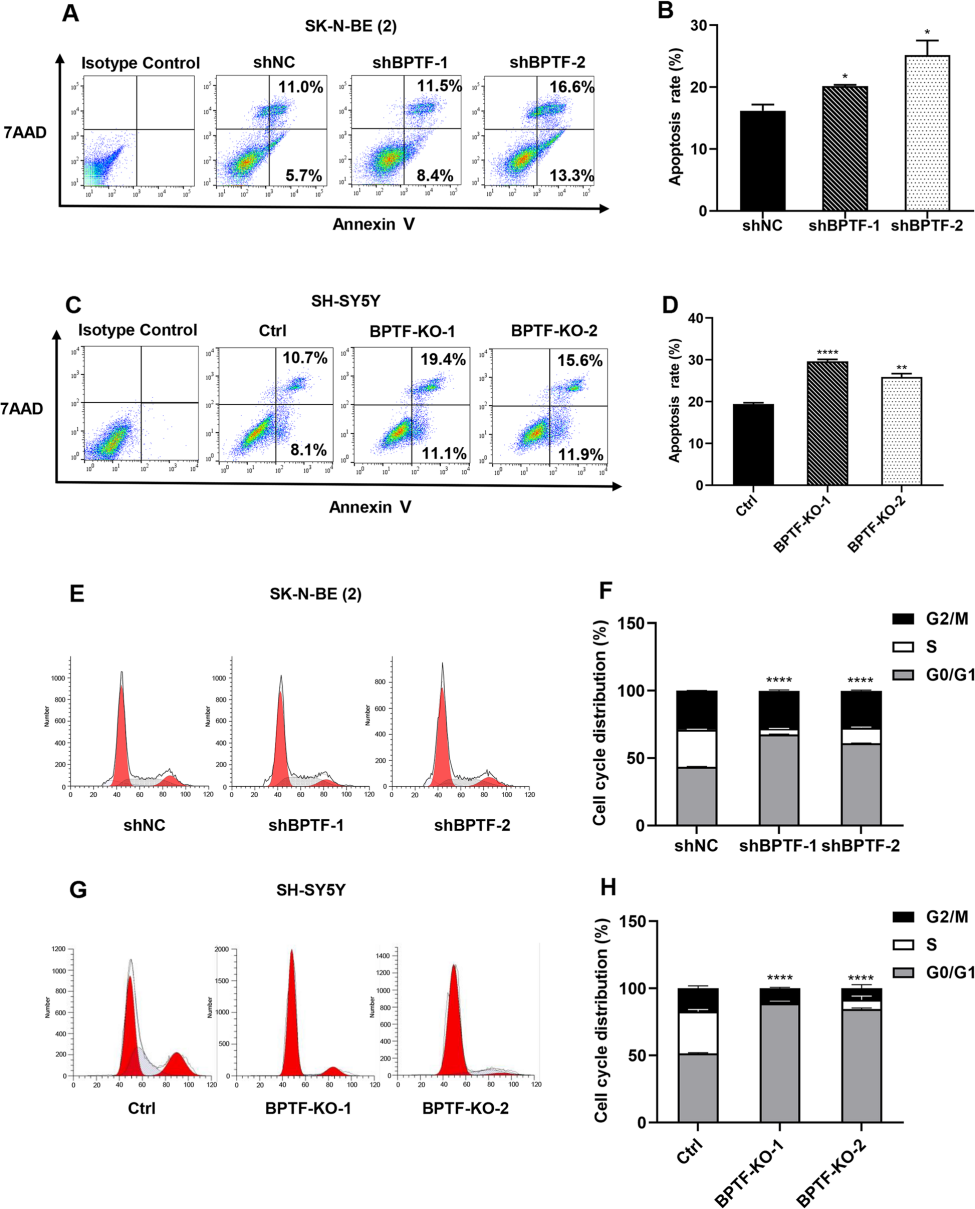
BPTF deficiency affects apoptosis and the cell cycle of NB cell. **A**, **C** SK-N-BE (2) cells [mean, 16.2% vs. 20.2% vs. 25.2%, A] and SH-SY5Y cells [mean, 19.4% vs. 29.6% vs. 25.9, C] were analyzed by flow cytometry for Annexin V and 7AAD dual labeling representatively. Annexin V-positive cells were designated as apoptotic cells. **B**, **D** The proportions of apoptotic SK-N-BE (2) cells (**B**) and SH-SY5Y cells (**D**). **E**, **G** The percentage of SK-N-BE (2) cells (**E**) and SH-SY5Y cells (**G**) in different phases of the cell cycle was determined by FACS analysis with PI staining. **F**, **H**) Distribution of Cell Cycle Phases in SK-N-BE (2) cells [G0/G1: mean, 43.6% vs. 67.4% vs. 61.0, F] and SH-SY5Y cells [G0/G1: mean, 51.5% vs. 89.0% vs. 84.7, H]. Error bars represent SEM. The data are representative of at least three independent experiments. * *P* < 0.05, **** *P* < 0.0001, as assessed by Student’s *t*-tests

To investigate whether BPTF affects NB proliferation through apoptosis, the effects of BPTF on apoptosis in NB cells were analyzed. BPTF knockdown SK-N-BE (2) cells and KO SH-SY5Y cells were found to show a higher proportion of Annexin V positive cells than negative control cells (Fig. 4A-D). It suggests that the lack of BPTF promotes apoptosis in NB cells. To confirm if BPTF was involved in mitotic progression, the cell cycle distribution in NB cell with BPTF silence was analyzed. The percentage of S-phase cells in the BPTF silence group was significantly lower than that in the control group (Fig. 4E-H). Altogether, these data revealed that high expression of BPTF could reduce apoptosis in NB cells and accelerate the cell cycle.

### BPTF activates the PI3K/AKT signaling pathway in NB cells

**Fig. 5:**
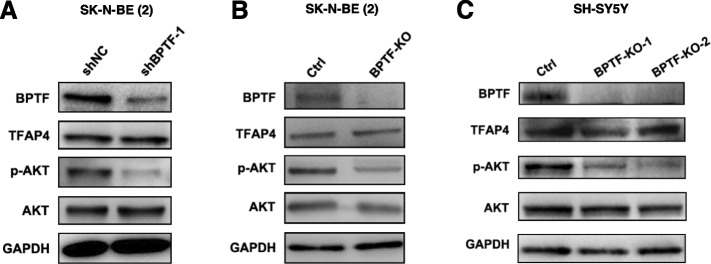
BPTF activates PI3K/AKT signaling pathway. **A** Representative western blot of PI3K/AKT molecule and TFAP4 in SK-N-BE (2) cells with BPTF knockdown. **B** Representative western blot of PI3K/AKT molecule and TFAP4 in SK-N-BE (2) cells with BPTF KO. **C** Representative western blot of PI3K/AKT molecule and TFAP4 in SH-SY5Y cells with BPTF KO. The data are representative of at least three independent experiments

It has been reported that PI3K/AKT signaling pathway is associated with proliferation and tumor cell metastasis in NB [23]. Our study showed that depletion of BPTF leads to down-regulate p-AKT expression in SK-N-BE (2) and SH-SY5Y cell lines separately (Fig. 5A-C). Taken together, our data demonstrated that BPTF functioned as an oncogene that promoted cell growth and migration/invasion capacity in NB by activating the PI3K/AKT pathway.

### BPTF is positively regulated by TFAP4 and promotes Epithelial–mesenchymal transition (EMT) process of NB cells through the activation of the PI3K/AKT signaling pathway

**Fig. 6:**
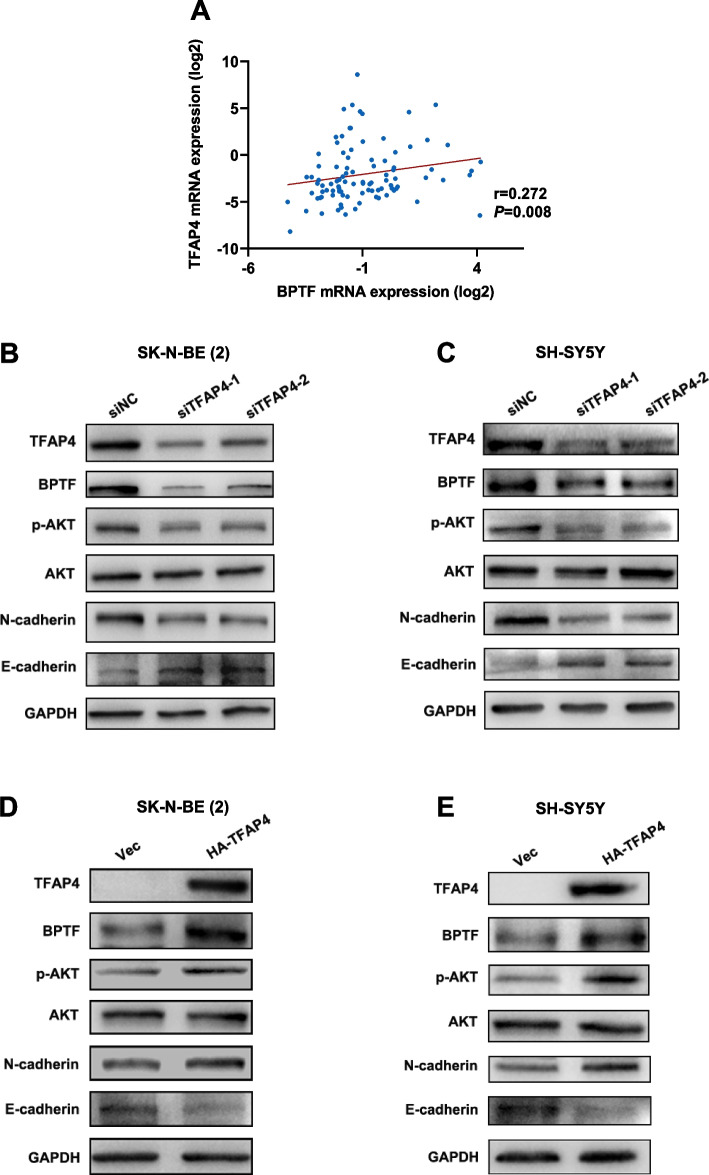
BPTF is positively regulated by TFAP4 and activates the downstream PI3K/AKT pathway. **A** PCR of the enrolled children with NB revealed a positive association between BPTF and TFAP4 (n = 95). r = 0.272, *P* = 0.008, by Spearman’s correlation coefficient. **B**-**C** Representative expression levels of BPTF, p-AKT, AKT, N-cadherin, E-cadherin and GAPGH in the SK-N-BE (2) (**B**) and SH-SY5Y cells (**C**) transfected with siNC and siTFAP4. **D**-**E** Representative expression levels of BPTF, p-AKT, AKT, N-cadherin, E-cadherin and GAPGH in the SK-N-BE (2) (**D**) and SH-SY5Y cells (**E**) cell transfected with Vec and HA-TFAP4 plasmid. The data are representative of at least three independent experiments

Predictive analysis of protein interactions with the BioGRID database (https://thebiogrid.org/108481/summary/homo-sapiens/bptf.html) and the STRING database (https://cn.string-db.org/cgi/network?taskId=bfr6S5fc1OT2&sessionId=bTGyXynR6Rp1) showed that TFAP4 was one of the higher-ranked related molecules in the BPTF protein association network (Fig. S3A). Studies have suggested that TFAP4 may play an oncogenic role in *MYCN*-amplified NB [14]. However, in this study, co-IP was performed and failed to verify the interaction between BPTF and TFAP4 (data not show). Meanwhile, even if there was no interaction between the two at the protein level, the expression of BPTF and TFAP4 were significantly positively correlated in NB by analysis of the GSE62564 dataset (*r* = 0.364, *P* < 0.001, Fig. S3B). The clinical cohort study of our center also confirmed the above results (*r* = 0.272, *P* = 0.008, Fig. 6A).

In different NB cell lines, BPTF, p-AKT, and N-cadherin in TFAP4-knockdown groups were significantly lower than control cells. Meanwhile, E-cadherin had the opposite trend in the same expriement (Fig. 6B-C). Conversely, BPTF and N-cadherin expression in TFAP4 overexpression cells were significantly increased, and E-cadherin was decreased and AKT phosphorylation was activated (Fig. 6D-E). However, no significant changes in TFAP4 after altering BPTF (Fig. 5A-B). Meanwhile, database indicated that BPTF were positively correlated with the levels of EMT markers, which could confirm these results (Fig. S3C-D). All of the above suggested that TFAP4 could positively regulate the expression of BPTF and then activate PI3K/AKT signaling pathway to induce the EMT process, which contributed to promoting the proliferation and metastasis of NB cells.

### Patient characteristics

To further explore the unique prognostic and potential therapeutic value of BPTF in NB, BPTF mRNA levels in the BM of 109 newly diagnosed children derived from BCH were detected. The study population consisted of 53 male (48.6%) and 56 femal (51.4%) with a median age of 41.7 (IQR, 24.25–65.45) months. Of these, 54 (49.5%) children were stage IV, 55 (50.5%) children were HR group and 49 (45%) children had BM metastasis. There were 15 (13.8%), 25 (23.6%) and 34 (32.1%) children with *MYCN* amplification, 1p36 deletion, and 11q23 deletion, respectively (Table 1).

**Table 1:** Characteristics at diagnosis of 109 patients with NB

Characteristic	Total	High BPTF	Low BPTF	χ^2^	*P*-values
Percent of sample, n (%)		55 (50.5)	54 (49.5)		
Sex, *n* (%)				3.305	0.069
Male	53 (48.6)	22 (40.0)	31 (57.4)		
Female	56 (51.4)	33 (60.0)	23 (42.6)		
Age (months), n (%)				1.155	0.283
< 18	18 (16.5)	7 (12.7)	11 (20.4)		
≥ 18	91 (83.5)	48 (87.3)	43 (79.6)		
Staging, *n* (%)				6.694	0.010*
I-III/IVs	55 (50.5)	21 (38.2)	34 (63.0)		
IV	54 (49.5)	34 (61.8)	20 (37.0)		
Risk group, *n* (%)				5.731	0.017*
LR or IR	54 (49.5)	21 (38.2)	33 (61.1)		
HR	55 (50.5)	34 (61.8)	21 (38.9)		
*MYCN* status, *n* (%)				0.057	0.810
Amplification	15 (13.8)	8 (14.5)	7 (13.0)		
Not amplification	94 (86.2)	47 (85.5)	47 (87.0)		
1p36 status, *n* (%)				0.052	0.819
LOH	25 (23.6)	13 (24.5)	12 (22.6)		
No loss	81 (76.4)	40 (75.5)	41 (77.4)		
11q23 status, *n*(%)				2.771	0.096
LOH	34 (32.1)	21 (39.6)	13 (24.5)		
No loss	72 (67.9)	32 (60.4)	40 (75.5)		
*PHOX2B*, *n* (%)				3.303	0.069
= 0	51 (46.8)	21 (38.2)	30 (55.6)		
> 0	58 (53.2)	34 (61.8)	24 (44.4)		
LDH (U/L), *n* (%)				1.216	0.749
≤ 295	36 (33.0)	18 (32.7)	18 (33.4)		
295–500	24 (22.0)	10 (18.2)	14 (25.9)		
500–1500	32 (29.4)	18 (32.7)	14 (25.9)		
> 1500	17 (15.6)	9 (16.4)	8 (14.8)		
NSE (ng/l), *n* (%)				4.863	0.088
≤ 25	26 (23.9)	12 (21.8)	14 (25.9)		
25–100	24 (22.0)	8 (14.5)	16 (29.7)		
> 100	59 (54.1)	35 (63.7)	24 (44.4)		
Primary tumor site, *n* (%)				6.161	0.013*
Retroperitoneum and adrenal glands	71 (65.1)	42 (76.4)	29 (53.7)		
Mediastinum/Pelvic cavity/Neck	38 (34.9)	13 (23.6)	25 ((46.3)		
Tumor size (cm), *n* (%)				6.262	0.044*
≤ 5	26 (23.9)	9 (16.4)	17 (31.5)		
5–10	43 (39.4)	20 (36.4)	23 (42.6)		
> 10	40 (36.7)	26 (47.2)	14 (25.9)		
Number of organs with metastasis, *n* (%)				2.711	0.100
≤ 3	60 (55.0)	26 (47.3)	34 (63.0)		
> 3	49 (45.0)	29 (52.7)	20 (37.0)		
BM metastasis, *n* (%)					
Yes	49 (45.0)	30 (54.5)	19 (35.2)	4.127	0.042*
No	60 (55.0)	25 (45.5)	3535353564.8)		

*LR* Low-risk, *IR* Intermediate-risk, *HR* High-risk, *MYCN* amplification of the *MYCN* gene, *LDH* Lactate dehydrogenase, *NSE* Neuron-specific enolase, *LOH* Loss of heterozygosity, *BM* Bone marrow. ^*^ P < 0.05, as assessed by Chi-square test

### BPTF is highly expressed in NB BM and correlated with clinical progression

**Fig. 7:**
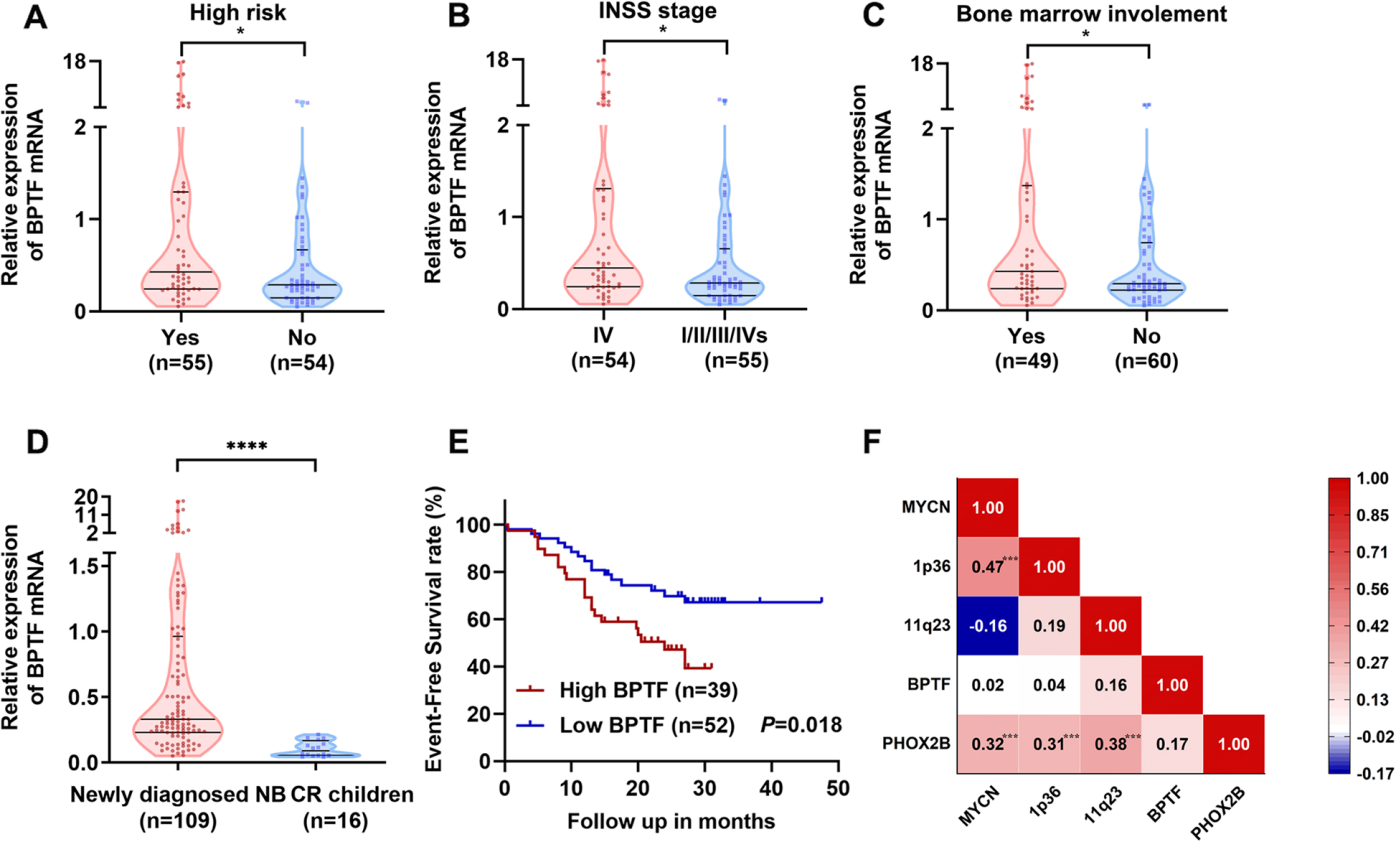
BPTF is highly expressed in NB bone marrow and correlated with clinical progression. **A** The expression of BPTF was significantly higher in the HR group than in non-high risk [median, 0.428 vs. 0.288, *n* = 109]. **B** The expression of BPTF was significantly higher in stage IV children than in stage I/II/III/IVs children [median, 0.446 vs. 0.283, *n* = 109]. **C** The expression of BPTF in the group with bone marrow metastasis was significantly higher than that in the group without bone marrow metastasis [median, 0.428 vs. 0.293, *n* = 109]. **D** BPTF in children with newly diagnosed NB was significantly higher than that in children with complete remission [median, 0.331 vs. 0.089, *n* = 125]. **E** Among the children followed up for more than 15 months, the children with high BPTF NB had a poor prognosis [3-year EFS, 39.3% vs. 67.2%, *n* = 91]. *P* = 0.018, by log-rank test. **F** Correlation analysis of the five genetic variables (*n* = 109), by Spearman’s correlation coefficient. * *P* < 0.05, *** *P* < 0.001, **** *P* < 0.0001, as assessed by Mann Whitney test

Patients were divided into high BPTF expression group and low expression group based on the median expression of BPTF [0.331 (IQR, 0.229–0.962)]. BPTF expression was significantly higher in HR group [median, 0.428 vs. 0.288 (non-high-risk group), *P* = 0.017], stage IV group [median, 0.446 vs. 0.283 (I-III / IVs group), *P* = 0.012], and NB children with BM metastasis group [median, 0.428 vs. 0.293 (non-BM metastases group), *P* = 0.046, Fig. 7A-C]. Compared with the 16 children with CR for at least 3 years, the newly diagnosed children had significantly higher BPTF expression in BM [median, 0.331 vs. 0.089, *P* < 0.0001, Fig. 7D].

The general clinical characteristics and distribution of the enrolled children are shown in Table 1. More children had high BPTF expression in stage IV group than in non-stage IV children (61.8% vs. 38.2%, *P* = 0.010), and in HR group than in non-HR children group (61.8% vs. 38.2%, *P* = 0.017), Retroperitoneum/adrenal primary tumor site had statistically significantly greater proportion of high BPTF expression than all other primary sites combined (76.4% vs. 23.6%, *P* = 0.013). More children had high BPTF expression in group with large primary tumor than in group with small size (47.2% vs. 36.4% vs. 16.4%, *P* = 0.044). Compared with children without BM metastasis, the proportion of high BPTF expression was significantly higher in children with BM metastasis (54.5% vs. 45.5%, *P* = 0.042).

A total of 91 children with NB were followed up for more than 15 months. Follow-up was completed through September 31, 2021. The median follow-up was 23 (IQR, 13.0–27.5) months. As shown in Fig. 7E, the 3-year EFS of high BPTF expression group was significantly lower than low BPTF expression group (39.3% vs. 67.2%, *P* = 0.018). Multivariate analysis showed that high BPTF expression (HR = 2.160, 95% CI = 1.121 to 4.161, *P* = 0.020), *MYCN* amplification (HR = 3.726, 95% CI = 1.793 to 7.740, *P* < 0.001) and 11q23 deletion (HR = 2.976, 95% CI = 1.550 to 5.712, *P* = 0.001) were independent prognostic factors associated with poor survival respectively (Table 2, Fig. 7F).

**Table 2:** Univariate and multivariate analysis of event‐free survival in 91 children with NB

Variable	Univariate		Multivariate	
	HR (95%CI)	*P*-values	HR (95%CI)	*P*-values
Sex				
male	ref		ref	
female	1.245 (0.652–2.378)	0.506	1.235 (0.618–2.466)	0.550
Age (months)				
< 18	ref		ref	
≥ 18	5.383 (1.293–22.412)	0.021*	2.483 (0.573–10.762)	0.224
BPTF				
< 0.331	ref		ref	
≥ 0.331	2.160 (1.121–4.161)	0.021*	2.349 (1.146–4.813)	0.020*
*MYCN* status				
Amplification	3.726 (1.793–7.740)	< 0.001*	4.408 (2.002–9.705)	< 0.001*
Not amplification	ref		ref	
11q23 status				
LOH	2.976 (1.550–5.712)	0.001*	3.068 (1.560–6.035)	0.001*
No loss	ref		ref	

*HR* Hazard ratios, *MYCN* amplification of the *MYCN* gene, *LOH* Loss of heterozygosity. ^*^ P < 0.05, as assessed by COX regression

### BPTF promotes progression of NB in vivo

**Fig. 8:**
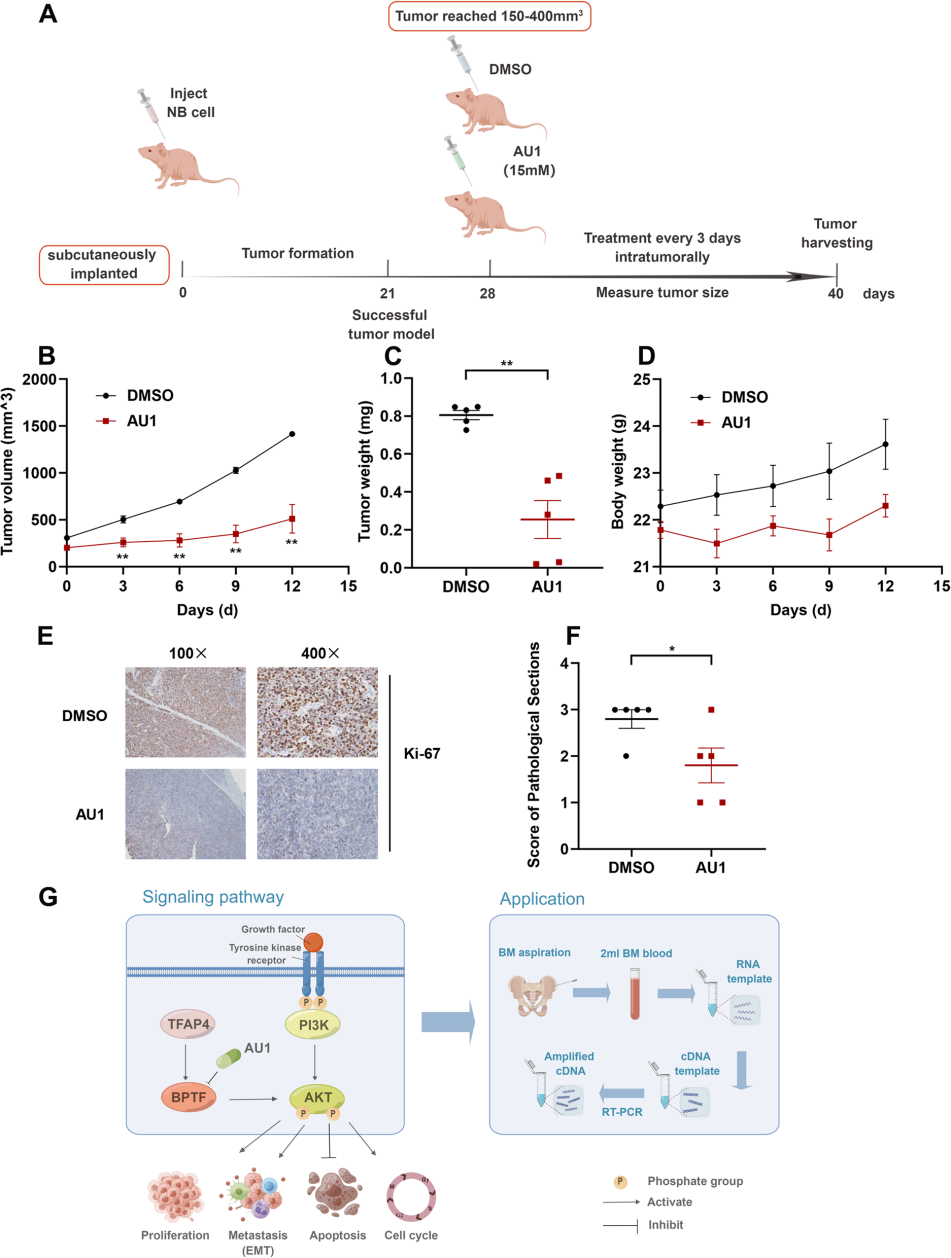
Inhibition of NB progression after treatment of AU1 in vivo. **A** The workflow of animal experiment. **B**-**C** Weight and volume of tumor for nude mice treated intratumorally with BPTF inhibitor AU1 and DMSO at the indicated time points (15 mM, 15ul/animal/3 days; data from 5 mice/group). **D** Body weight of mice during treatment [mean, 0.805 vs. 0.255]. **E** Representative images of immunostaining of Ki67 in tumor sections. **F** The statistical graph of IHC [mean, 2.800 vs. 1.800]. **G** Schematic diagram showing that BPTF is regulated by TFAP4, activates the PI3K/AKT pathway to promote NB progression, and its clinical application. Error bars represent SEM. * *P* < 0.05, ** *P* < 0.01, as assessed by Student’s *t*-tests

To investigate the progression-promoting roles of BPTF in NB, its inhibitor AU1 was used following the workflow (Fig. 8A). As shown in Fig. 8B-C, tumor sizes and weights in the AU1 group were significantly lower than those in the DMSO group. AU1 treatment resulted in no statistically significant changes in body weight compared with that of DMSO (Fig. 8D). Next, IHC results signified that BPTF silence could reduce Ki-67 positive cells in the tumors of mice (Fig. 8E-F). The above indicated that treatment of BPTF inhibitor AU1 could prevent NB progression in vivo. Moreover, AU1 might act as a drug targeting BPTF for treating NB.

## Discussion

In recent years, the application of BM detection in the early diagnosis of NB has become increasingly wide and necessary [6]. It can detect tumor residues imperceptible by imaging examination and indicate prognosis. Our study suggested that BPTF has an oncogenic function for NB and quantification of BPTF mRNA in BM can predict NB progression independently.

BPTF is an emerging epigenetic regulatory protein. As the largest subunit of NURF, BPTF plays a crucial role in the formation of NURF and the maintenance of its functional activity [24]. NURF is a complex that changes chromatin dynamics to control transcription and chromatin remodeling [25]. Recent studies on chromatin remodeling and tumors have been in the ascendant, especially targeted therapy based on chromatin regulators has become a promising therapeutic strategy [26]. Among them, the relationship between BPTF and tumor development has been gradually revealed. Studies have reported that BPTF overexpression can predict poor prognosis in various malignant tumors, such as melanoma, colon cancer and non-small-cell-type lung cancer [27–29]. It also can promote the metastasis of colon cancer [30], non-small cell lung cancer [31] and renal cell carcinoma [32] through EMT, c-MYC and METTL14, respectively.

This study firstly analyzed database and found that the expression level of BPTF was related to the OS and EFS of children with NB, although not statistically significant in EFS. The main reason for the insignificant EFS *P*-value was that the cohort was performed with tumor tissue, and the follow-up time in most patients was short. The difference between the two groups might gradually increase with the extension of the follow-up time. Furthermore, when BPTF was silenced or BPTF inhibitor AU1 was used, the proliferation and metastasis of NB cells were decreased in vitro and in vivo. In addition, the cell cycle was blocked, and the apoptosis was increased in BPTF-depleted NB cells. Thus, BPTF was an oncogenic molecule of NB and might be a potential target for NB diagnosis and treatment. AU1 may play a role in NB as a drug targeting BPTF.

Prompted by the database, it was predicted a positive correlation between BPTF and TFAP4. TFAP4 is involved in the proliferation, metastasis, differentiation and angiogenesis of various tumors, including NB [33–35]. A study reported the presence of the *MYCN*-TFAP4 axis in NB with *MYCN* amplification and identified potential therapeutic targets for this aggressive form of the disease [36]. However, the relationship between BPTF and TFAP4 has not yet been revealed. TFAP4 can activate PI3K/AKT signaling to promote HCC invasion and metastasis through the induction of EMT [37, 38]. Multiple driver genes affected the proliferation and transfer of NB through the PI3K/AKT pathway, including Speedy/RINGO, XPO1, etc. [39, 40]. EMT has been reported to be closely associated with tumor metastasis, which gives NB cells an aggressive phenotype with increased metastatic potential [41].

Inspired by this, our study showed that BPTF silence could down-regulate the expression of p-AKT, which represented the PI3K/AKT was inhibited. It was further found that when TFAP4 was overexpressed, not only BPTF expression was increased, but also p-AKT was inhibited, and EMT pathway-related molecules also changed accordingly. In contrast, alterations in BPTF were unable to cause significant changes in TFAP4. It hinted that BPTF was a downstream molecule of TFAP4 in signal pathway. However, BPTF did not directly interact with the TFAP4 at the protein level, which was detected by co-IP (data not show). All of the above demonstrated that TFAP4 could positively regulate the expression of BPTF and activate the downstream PI3K/AKT pathway. Thereby BPTF could cause the EMT process to promote the proliferation and metastasis of NB. The above further revealed the tumor-promoting molecular mechanism of BPTF in NB cells.

Collectively, these functional and mechanistic experiments hinted that BPTF detection might be futher applicated in BM of NB patient to predict clinical prognosis. Furthermore, in order to confirm this hypothesis in real-world clinical settings, BM specimens from 109 children with NB in BCH were examined. BM is the most common site of infiltration in NB with metastatic disease and also prone to disease recurrence [42]. The persistence of disease in the BM predicts a poor prognosis. BM testing has been recommended by the International Neuroblastoma Response Criteria Bone Marrow Working Group (INRG BMWG) as a method to assess disease response [43]. It can overcome the disadvantage that NB tumor tissue is difficult to gain before chemotherapy, especially HR-NB. BM examination can monitor MRD not detected by other conventional indicators in advance, realizing early diagnosis of NB progression [44]. RT-PCR has been introduced into clinical practice as an ideal tool for monitoring MRD with a sensitivity of 1 tumor cell in 10^5^ to 10^7^ normal cells [45]. A study applied RT-PCR to quantify the mRNA of five NB-related genes (*CHGA*, *DCX*, *DDC*, *PHOX2B*, and *TH*) and found that they were independently associated with EFS in relapsed/refractory NB [46].

In our study, RT-PCR was used to detect the mRNA level of BPTF in BM, which is more convenient and sensitive for clinical application. Statistical analysis of the 109 NB cohorts in our center suggested that the HR group, stage IV and NB children with BM metastasis had higher expression levels of BPTF than the other group. Especially, samples were taken with BM specimens, which could be obtained before the first chemotherapy and surgery. Although this was a pool study, BPTF could be regarded as a molecule predicting minimal residues in the BM. This was reflected in the fact that the mRNA level of BPTF in newly diagnosed children was significantly higher than that in CR children. EFS was followed up as the primary index for evaluating tumor progression and recurrence. The EFS of high BPTF group was significantly lower than that of low BPTF group. At present, studies have found that factors such as *MYCN* amplification [47], 1p36 [7] and 11q23 [48] loss in BM are associated with poor prognosis in NB. Univariate and multivariate analysis in our study also proved that BPTF was an risk factor for NB recurrence and progression independent of other clinical factors (*MYCN* amplification and 11q23 loss). Therefore, detection of BPTF in BM can effectively and independently predict the outcome of NB, which is helpful for early clinical diagnosis. As a potential progression target of NB, BPTF and its inhibitor AU1 were both worthy of further application. In the future, we will continue to monitor the expression level of BPTF in the BM from children with NB in different treatment procedures to clarify its dynamic detection capability.

## Conclusions

In summary, this study confirms that BPTF is highly expressed in NB and can be as a new independent risk factor affecting the prognosis of NB. BPTF regulated by TFAP4 positively activates the PI3K/AKT pathway and induces cells to undergo EMT, which then promotes the proliferation and metastasis ability of NB cells. And ultimately leads to NB recurrence and progression. As a biological target independent of *MYCN* amplification and 11q23 loss, BPTF in BM is a potential progression and therapeutic target for NB. BPTF inhibitor may have important theoretical significance and potential applications for the treatment of NB (Fig. 8G).

## Supplementary Information

**Additional file 1: Figure S1.** **A** GFP fluorescence imaging of 2 unique shRNA constructs targeting BPTF in SK-N-BE (2) cells 96 hours after lentiviral infection, when observed best transfection efficiency. Scale bar is 50 μm. **B** Quantitative of BPTF after 96h infection with BPTF knockdown lentiviral, as detected by RT-PCR [mean, 1.222 vs. 0.845 vs. 0.972]. **C** Sequencing results of the BPTF KO cell in SK-N-BE (2) compared with Ctrl. **D** Western blot of BPTF in SK-N-BE (2) cell with BPTF KO [mean, 0.386 vs. 0.000]. **E**, **G** Sequencing results of the BPTF KO cell in SH-SY5Y compared with Ctrl. **F**, **H** Representative western blot of BPTF in SH-SY5Y cell [mean, 0.442 vs. 0.000, D; 0.225 vs. 0.000, F] with BPTF KO. Error bars represent SEM. The data are representative of at least three independent experiments. * *P*<0.05, ** *P*<0.01, **** *P*<0.0001, as assessed by Student’s t-tests.  

**Additional file 2: Figure S2. A **SH-SY5Y cell growth following BPTF KO shown by colony formation assay [mean, 114.330 vs. 81.330 vs. 45.000]. Error bars represent SEM.** B **SH-SY5Y cell migration following BPTF KO shown by RTCA. Error bars represent SD. The data are representative of at least three independent experiments. * *P*<0.05, ** *P*<0.01, as assessed by Student’s t-tests..

**Additional file 3: Figure S3. A **The STRING database hinted that TFAP4 was one of the higher-ranked related molecules associated with BPTF.** B **The GEO NB cohorts showed that the expression of BPTF and TFAP4 were significantly positively correlated (*n*=498). *r*=0.211, *P*<0.0001.** C **Correlation analysis between BPTF and N-cadherin from the GEO database (*n*=498). *r*=0.147, *P*=0.001.** D **Correlation analysis between BPTF and E-cadherin from the GEO database (*n*=498). *r*=-0.199, *P*=0.008. They all assessed by Spearman’s correlation coefficient.**.**

## Data Availability

Datasets and other files generated or analyzed during this study are included in this article and its supplementary files. More data are available from the corresponding author upon reasonable request.

## Abbreviations

BPTF: Bromodomain PHD finger transcription factor
BM: Bone marrow
NB: Neuroblastoma
EMT: Epithelial-mesenchymal transition
HR: High-risk
TFAP4: Transcriptional factor activating protein 4
INSS: International Neuroblastoma Stage System
COG: Children’s Oncology Group
MRD: Minimal residual disease
CR: Completely remission
KO: Knock-out
RT-PCR: Real-time PCR
RTCA DP: Real-Time Cell Analysis Dual Purpose
SEM: Standard error
SD: Standard deviation
EFS: Event-free survival
HR: Hazard ratios
CI: Confidence interval
INRG BMWG: International Neuroblastoma Response Criteria Bone Marrow Working group
LR: Low-risk
IR: Intermediate-risk
*MYCN*: Amplification of the *MYCN* gene
LDH: Lactate dehydrogenase
NSE: Neuron-specific enolase
LOH: Loss of heterozygosity
