# Supplementary figures and images for: BPTF in bone marrow provides a potential progression biomarker regulated by TFAP4 through the PI3K/AKT pathway in neuroblastoma
Source: Biol Proced Online. 2023 May 11;25:11. doi: 10.1186/s12575-023-00200-7 (PMC10176855; doi:10.1186/s12575-023-00200-7)

Figure S1.

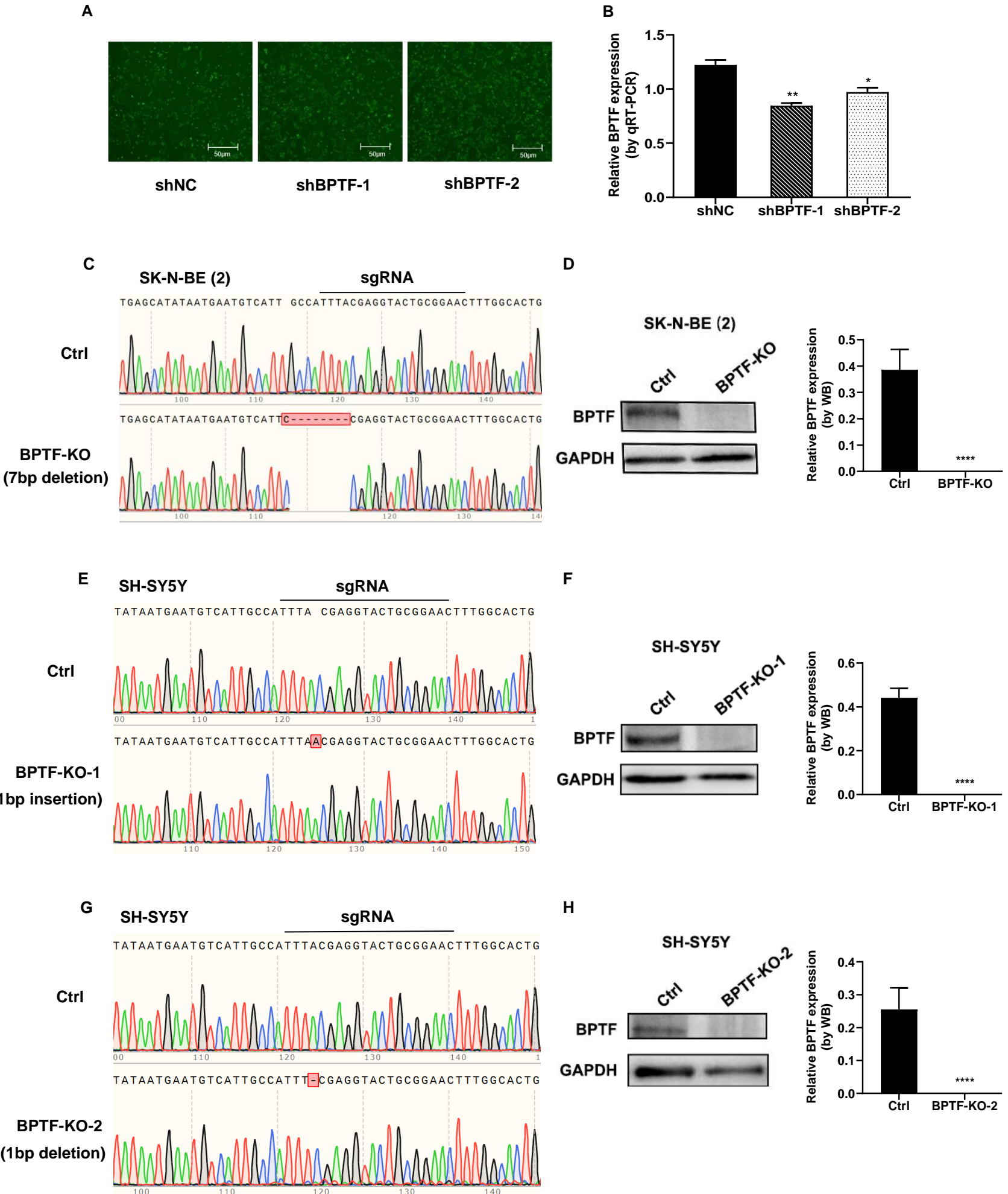

Supplement: Supplementary file 1 — Additional file 1: Figure S1. A GFP fluorescence imaging of 2 unique shRNA constructs targeting BPTF in SK-N-BE (2) cells 96 hours after lentiviral infection, when observed best transfection efficiency. Scale bar is 50 μm. B Quantitative of BPTF after 96h infection with BPTF knockdown lentiviral, as detected by RT-PCR [mean, 1.222 vs. 0.845 vs. 0.972]. C Sequencing results of the BPTF KO cell in SK-N-BE (2) compared with Ctrl. D Western blot of BPTF in SK-N-BE (2) cell with BPTF KO [mean, 0.386 vs. 0.000]. E, G Sequencing results of the BPTF KO cell in SH-SY5Y compared with Ctrl. F, H Representative western blot of BPTF in SH-SY5Y cell [mean, 0.442 vs. 0.000, D; 0.225 vs. 0.000, F] with BPTF KO. Error bars represent SEM. The data are representative of at least three independent experiments. * P<0.05, ** P<0.01, **** P<0.0001, as assessed by Student’s t-tests. [file 12575_2023_200_MOESM1_ESM.pdf]

Figure S2.

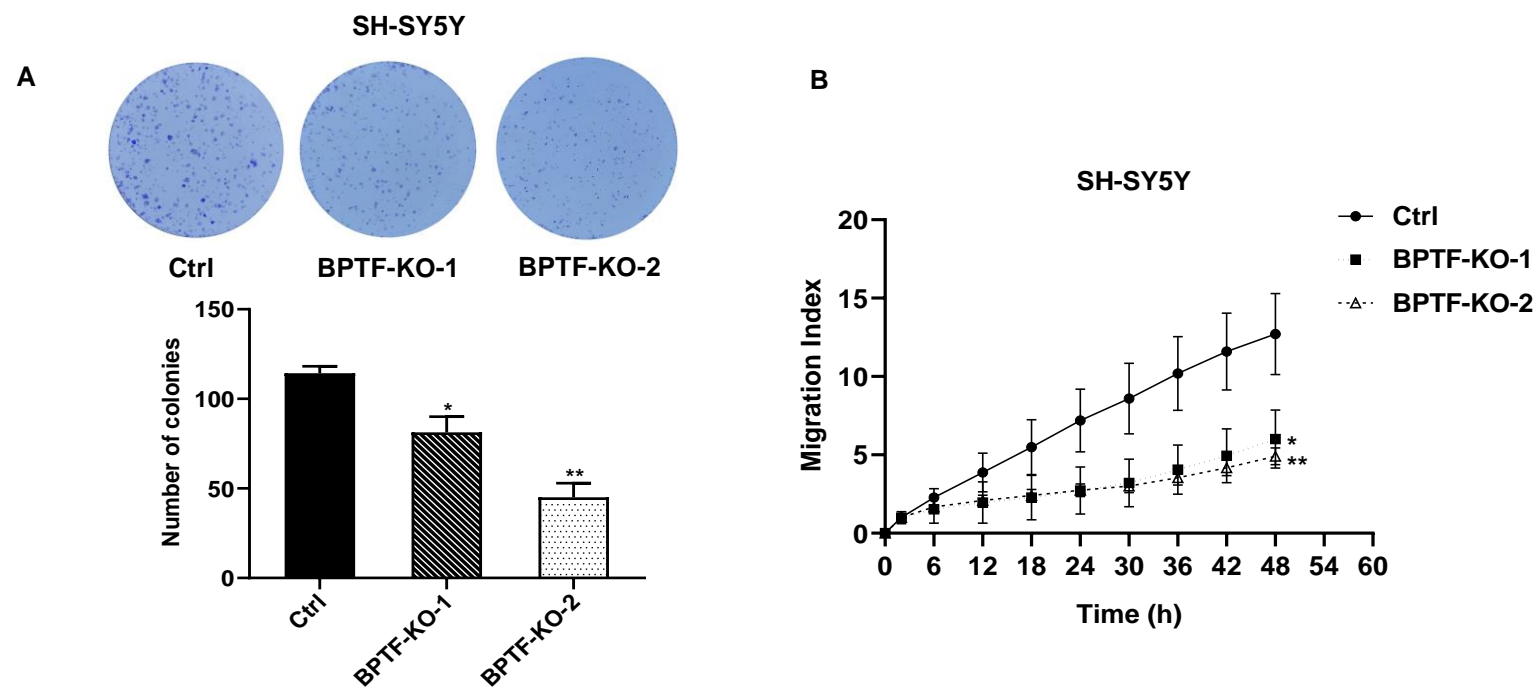

Supplement: Supplementary file 2 — Additional file 2: Figure S2. A SH-SY5Y cell growth following BPTF KO shown by colony formation assay [mean, 114.330 vs. 81.330 vs. 45.000]. Error bars represent SEM. B SH-SY5Y cell migration following BPTF KO shown by RTCA. Error bars represent SD. The data are representative of at least three independent experiments. * P<0.05, ** P<0.01, as assessed by Student’s t-tests.. [file 12575_2023_200_MOESM2_ESM.pdf]

Figure S3.

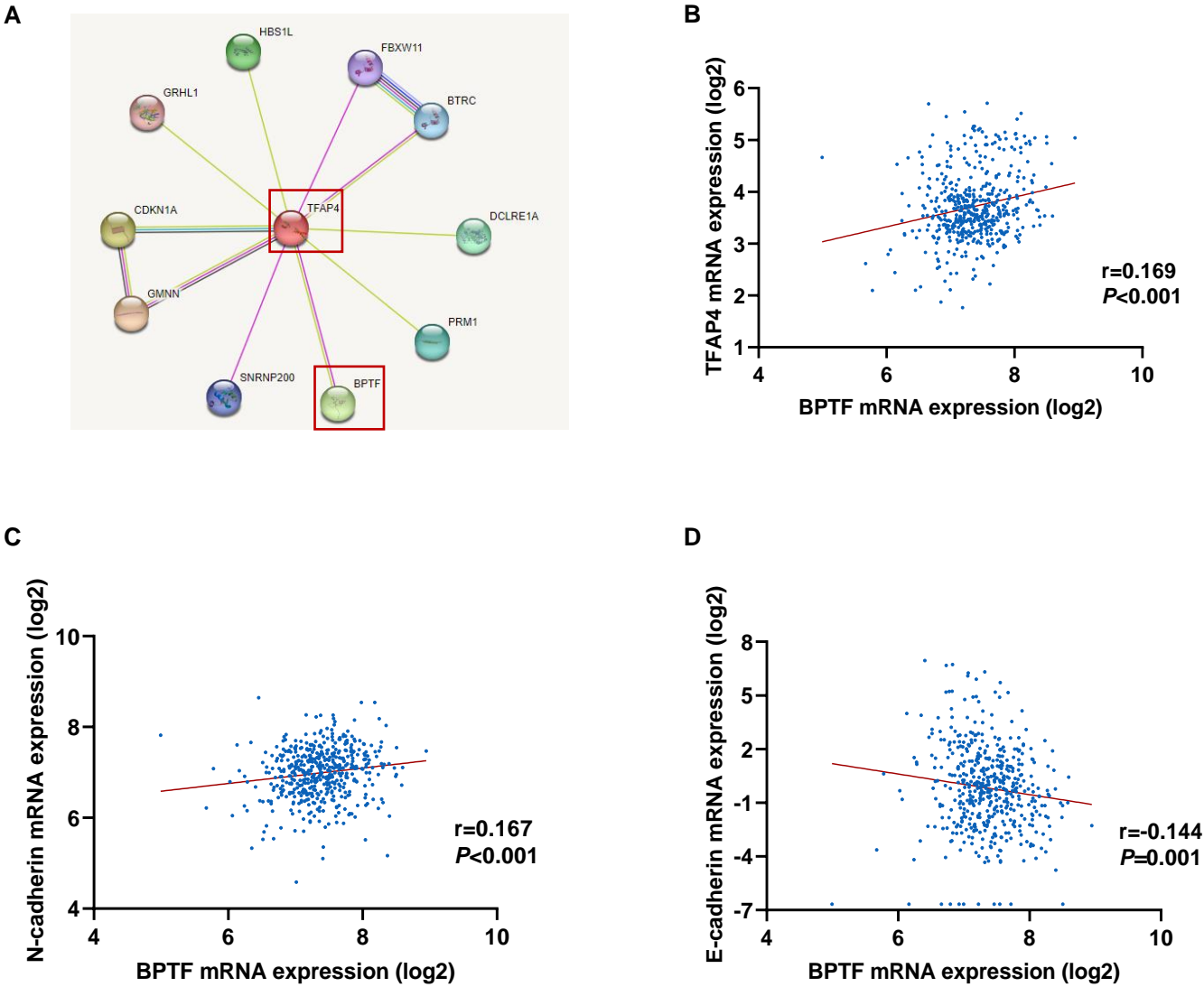

Supplement: Supplementary file 3 — Additional file 3: Figure S3. A The STRING database hinted that TFAP4 was one of the higher-ranked related molecules associated with BPTF. B The GEO NB cohorts showed that the expression of BPTF and TFAP4 were significantly positively correlated (n=498). r=0.211, P<0.0001. C Correlation analysis between BPTF and N-cadherin from the GEO database (n=498). r=0.147, P=0.001. D Correlation analysis between BPTF and E-cadherin from the GEO database (n=498). r=-0.199, P=0.008. They all assessed by Spearman’s correlation coefficient.. [file 12575_2023_200_MOESM3_ESM.pdf]
